# Risk factors for late defecation and its association with the outcomes of critically ill patients: a retrospective observational study

**DOI:** 10.1186/s40560-016-0156-1

**Published:** 2016-04-29

**Authors:** Shinya Fukuda, Takashi Miyauchi, Motoki Fujita, Yasutaka Oda, Masaki Todani, Yoshikatsu Kawamura, Kotaro Kaneda, Ryosuke Tsuruta

**Affiliations:** Advanced Medical Emergency and Critical Care Center, Yamaguchi University Hospital, 1-1-1 Minami-Kogushi, Ube, Yamaguchi 755-8505 Japan; Acute and General Medicine, Yamaguchi University Graduate School of Medicine, Ube, Yamaguchi Japan

**Keywords:** Constipation, Intensive care unit, Sedatives, Enteral nutrition, C-reactive protein

## Abstract

**Background:**

Late defecation was recently reported to be associated with worse clinical outcomes in critically ill patients. However, more research is needed to examine the causes and clinical significance of late defecation. The objectives of this study were to investigate the risk factors for late defecation and its association with the outcomes of intensive care unit (ICU) patients.

**Methods:**

Patients in an ICU for ≥7 days between January and December 2011 were retrospectively assessed. Based on the time between admission and the first defecation, they were assigned to early (<6 days; *n* = 186) or late (≥6 days; *n* = 96) defecation groups. Changes in clinical variables between admission and ICU day 7 were assessed to investigate the effects of late defecation. The clinical outcomes were ICU mortality, length of ICU stay, and length of mechanical ventilation.

**Results:**

Late enteral nutrition (odds ratio (OR) 3.42; 95 % confidence interval (CI) 1.88–6.22; *P* < 0.001), sedatives (OR 3.07; 95 % CI 1.71–5.52; *P* < 0.001), and surgery (OR 1.86; 95 % CI 1.01–3.42; *P* = 0.047) were the independent risk factors for late defecation. The median (interquartile) changes in body temperature (0.3 [−0.4 to 1.0] vs 0.7 [0.1 to 1.5] °C; *P* = 0.004), serum C-reactive protein concentration (1.6 [−0.5 to 6.6] vs 3.5 [0.7 to 8.5] mg/dL; *P* = 0.035), and Sequential Organ Failure Assessment score (−1 [−2 to 1] vs 0 [−1 to 2]; *P* = 0.008) between admission and ICU day 7 were significantly greater in the late defecation group than in the early defecation group. ICU stay was significantly longer in the late defecation group (12 [9 to 19] vs 16 [10 to 23] days; *P* = 0.021), whereas ICU mortality and the length of mechanical ventilation were similar in both groups.

**Conclusions:**

Late enteral nutrition, sedatives, and surgery were independent the risk factors for late defecation in critically ill patients. Late defecation was associated with prolonged ICU stay.

## Background

Constipation is one of the many gastrointestinal problems that occur in critically ill patients. Mostafa et al. reported that the frequency of constipation was as high as 83 % in intensive care units (ICUs) [1]. The time from admission to the first defecation was 4.8 days in an ICU setting [2]. It was also reported that the time to the first defecation was almost 6 days in mechanically ventilated patients [3, 4]. Previous studies have shown that constipation is associated with difficulties in ventilator weaning [1, 5], increased infection rates [3], attenuated organ function [3, 4], increased ICU stay, and increased ICU mortality [3, 4, 6].

Previous studies have reported that constipation was attributed to various factors, including immobility [7], neurogenic imbalance [8], hypotension, hypoxemia [3], and the administration of opioids [9] and vasopressors [10]. In this study, we explored the issue of constipation in critically ill patients. The objectives of this study were to investigate the risk factors for late defecation and its association with the outcomes of critically ill patients.

## Methods

### Study design

This study was a retrospective single-center analysis conducted in a 20-bed ICU of a university hospital in Yamaguchi Prefecture, Japan. The study was approved by the Institutional Review Board of Yamaguchi University Hospital, and information about this study was published on the hospital’s Web page until August 2013.

### Patient selection

Adults (≥18 years old) treated in our ICU between January 1 and December 31, 2011, were screened for inclusion in this study. Patients who stayed in the ICU for <7 days were excluded. The other exclusion criteria were as follows: patients who presented with bloody stools, who had a permanent colostomy, who were mechanically ventilated for >2 days before admission, who underwent abdominal surgery immediately ≤7 days after admission, or who were withdrawn from aggressive treatment.

### Data collection

The following data were obtained from the patients’ medical records: age, sex, body mass index, Acute Physiology and Chronic Health Evaluation (APACHE) II score [11], main diagnosis, dates of admission and the first defecation, intervention to promote bowel elimination (such as picosulfate, magnesium oxide, lactulose, sennoside, bisacodyl suppository, sodium bicarbonate suppository, or glycerin enema), date of initial enteral nutrition, use of mechanical ventilation, use of medications, and type of major surgery. “Use of medications” was defined as the continuous administration of medications, such as sedatives, opioids, and vasopressors, for >24 h. “Use of mechanical ventilation” was defined as the use of a ventilator for >24 h after intubation. The length of ICU stay, the length of mechanical ventilation, and ICU mortality were recorded as the clinical outcomes. The data on body temperature, white blood cell count, serum C-reactive protein (CRP) concentration, and the Sequential Organ Failure Assessment (SOFA) score [12] on admission and ICU day 7 were also obtained from the patients’ medical records. In general, routine blood sampling and laboratory tests were performed daily in the ICU patients. If blood sampling was not performed on ICU day 7, data obtained on the closest day were used in the analysis.

### Definitions

Based on the previous study by van der Spoel et al. [4], early defecation was defined as defecation at ≤5 days after ICU admission and late defecation was defined as defecation ≥6 days after ICU admission. Late enteral nutrition was defined as the initiation of enteral nutrition ≥2 days after ICU admission according to the nutrition support therapy guideline established by the American Society for Parenteral and Enteral Nutrition (ASPEN) [13].

### Statistical analysis

Categorical variables are presented as numbers and percentages and were analyzed using the *χ*^2^ test. All continuous variables are presented as medians and interquartile ranges (IQRs), and the Mann–Whitney test was used to compare the two groups. Univariate and multivariate analyses using logistic regression with a stepwise selection procedure were performed to identify the variables associated with late defecation. The patients’ basic characteristics, APACHE II scores, and the other parameters with *P* values <0.10 in the univariate analysis were entered into the multivariate analysis.

All statistical analyses were performed with two-tailed tests, and *P* < 0.05 was considered significant. All analyses were performed with IBM SPSS Statistics for Windows version 19.0 (SPSS Inc., Chicago, IL).

## Results

**Fig. 1:**
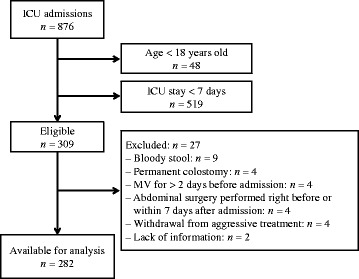
Patient selection and reasons for exclusion. *MV* mechanical ventilation, *ICU* intensive care unit

Of 876 patients admitted to the ICU, 309 were eligible for this study (Fig. 1). Twenty-seven patients were subsequently excluded owing to the exclusion criteria. Therefore, the data for 282 patients were available for analysis.

Based on the definitions of this study, the patients were divided into two groups: the early defecation group (*n* = 186, 66 %) or the late defecation group (*n* = 96, 34 %). There were no significant differences in the basic physiological characteristics or laboratory variables between the two groups, except for the serum CRP concentrations on admission (Table 1). The baseline diagnoses on admission were distributed similarly in both groups. In the total cohort of patients, the median (IQR) time between admission and the first defecation was 4 (3 to 6) days. Interventions to promote bowel elimination were performed significantly earlier in patients in the early defecation group than in patients in the late defecation group. The proportion of patients who received interventions was significantly smaller in the early defecation group (*n* = 93, 50 %) than in the late defecation group (*n* = 83, 86 %; *P* < 0.001). Enteral nutrition was initiated significantly earlier in the early defecation group than in the late defecation group. The proportion of patients who started enteral nutrition ≥2 days after admission (late enteral nutrition) was significantly smaller in the early defecation group (*n* = 85, 46 %) than in the late defecation group (*n* = 76, 79 %; *P* < 0.001).

**Table 1:** Patient characteristics

	Early defecation group		Late defecation group		*P* value
	(*n* = 186)		(*n* = 96)		
Age, years	68	(59–78)	70	(54–80)	0.96
Male, *n* (%)	107	(58)	51	(53)	0.48
BMI, kg/m^2^	21	(19–24)	22	(20–24)	0.34
Physiological and laboratory findings on admission					
Body temperature, °C	36.5	(36.0–37.2)	36.5	(35.8–36.9)	0.22
WBC count, ×1000/μL	11	(8–16)	11	(8–15)	0.87
Serum CRP, mg/dL	0.7	(0.1–6.0)	0.2	(0.1–0.7)	0.003
APACHE II score	13	(9–21)	16	(9–21)	0.67
SOFA score	4	(2–6)	4	(2–5)	0.14
Diagnosis on admission, *n* (%)					0.079
Cardiovascular disease	37	(20)	21	(22)	
Neurological disorder or stroke	60	(32)	46	(48)	
Infection	27	(15)	6	(6)	
External causes (e.g., trauma, intoxication, burn)	33	(18)	13	(14)	
Post cardiac arrest	11	(6)	4	(4)	
Others	18	(10)	6	(6)	
Time (days) from admission to the:					
First defecation	3	(2–4)	7	(6–8)	<0.001
First intervention to promote elimination	3	(2–4)	6	(4–7)	<0.001
Initiation of enteral nutrition	2	(2–3)	3	(3–5)	<0.001
Mechanical ventilation, *n* (%)	75	(40)	59	(61)	0.001
Sedative use, *n* (%)	73	(39)	71	(74)	<0.001
Fentanyl use, *n* (%)	24	(13)	28	(29)	0.001
Vasopressor use, *n* (%)	49	(26)	26	(27)	0.89
Surgery, *n* (%)	34	(18)	41	(43)	<0.001
Type of surgery, *n* (%)					0.26
Neurological	25	(74)	35	(85)	
Cardiovascular	1	(3)	2	(5)	
Orthopedic	5	(15)	1	(2)	
Debridement of skin and subcutaneous tissue	3	(9)	2	(5)	
Others	0	(0)	1	(2)	

All data are presented as the median (interquartile range) or *n* (%)

*BMI* body mass index, *WBC* white blood cell, *CRP* C-reactive protein, *APACHE II* Acute Physiology and Chronic Health Evaluation (second revision), *SOFA* Sequential Organ Failure Assessment

Mechanical ventilation was used for significantly more patients in the late defecation group than in the early defecation group. Sedatives and fentanyl (the only opioid used in this population of patients) were administered to significantly more patients in the late defecation group than in the early defecation group. The types of sedatives used for patients were similarly distributed in the early and late defecation groups: propofol, 44/73 (60 %) vs 49/71 (69 %), respectively; midazolam, 24/73 (33 %) vs 19/71 (27 %), respectively; others, 5/73 (7 %) vs 3/71 (4 %), respectively (*P* = 0.52). Among mechanically ventilated patients, sedatives were continuously administered to 62 (83 %) patients in the early defecation group and to 56 (95 %) patients in the late defecation group. Surgery was performed in significantly more patients in the late defecation group than in the early defecation group. The types of surgery were distributed similarly in both groups, and all surgeries were performed in an emergency situation, including 60 neurosurgeries for traumatic brain injury, stroke, subarachnoid hemorrhage, and brain herniation.

Univariate logistic regression analysis indicated that late enteral nutrition, mechanical ventilation, sedative use, fentanyl use, and surgery were associated with late defecation (Table 2). The multivariate model based on the variables selected in the univariate analysis revealed that late enteral nutrition, sedative use, and surgery were the independent risk factors for late defecation.

**Table 2:** Risk factors for late defecation

	Univariate		Multivariate	
	OR (95 % CI)	*P* value	OR (95 % CI)	*P* value
Age	1.00 (0.99–1.01)	0.94		
Male sex	0.84 (0.51–1.37)	0.48		
BMI	1.01 (0.97–1.06)	0.61		
Serum CRP	0.97 (0.93–1.00)	0.063		
APACHE II score	1.00 (0.98–1.03)	0.76		
SOFA score	0.93 (0.85–1.02)	0.108		
Diagnosis on admission		0.090		
Late enteral nutrition	4.52 (2.55–7.99)	<0.001	3.42 (1.88–6.22)	<0.001
Mechanical ventilation	2.36 (1.43–3.91)	0.001		
Sedative use	4.40 (2.56–7.56)	<0.001	3.07 (1.71–5.52)	<0.001
Fentanyl use	2.78 (1.50–5.14)	0.001		
Surgery	3.33 (1.92–5.77)	<0.001	1.86 (1.01–3.42)	0.047

*OR* odds ratio, *CI* confidence interval, *BMI* body mass index, *CRP* C-reactive protein, *APACHE II* Acute Physiology and Chronic Health Evaluation (second revision), *SOFA* Sequential Organ Failure Assessment

Changes in the patients’ physiological and laboratory parameters between admission and ICU day 7 were compared between the early and late defecation groups (Table 3). Although not all of the patients underwent blood sampling on day 7, the proportion of patients with blood samples obtained on day 7 was similar in the early (162/186, 87 %) and late defecation groups (86/96, 90 %; *P* = 0.70). The changes in body temperature, serum CRP concentrations, and SOFA scores were significantly greater in the late defecation group than in the early defecation group, whereas the white blood cell count was not significantly different between the two groups.

**Table 3:** Changes in physiological and laboratory variables between ICU day 1 and day 7

	Early defecation group		Late defecation group		*P* value
Body temperature, °C	0.3	(−0.4 to 1.0)	0.7	(0.1 to 1.5)	0.004
WBC count, ×1000/μL	−3.275	(−6.605 to 138)	−2.685	(−5.248 to 623)	0.29
Serum CRP, mg/dL	1.6	(−0.5 to 6.6)	3.5	(0.7 to 8.5)	0.035
SOFA score	−1	(−2 to 1)	0	(−1 to 2)	0.008

All data are presented as the median (interquartile range). The change in each variable was calculated as the value on day 7 minus the value on day 1

*ICU* intensive care unit, *WBC* white blood cell, *CRP* C-reactive protein, *SOFA* Sequential Organ Failure Assessment

The ICU mortality rate was not significantly different between the early and late defecation groups (Table 4). The length of ICU stay was significantly greater in the late defecation group than in the early defecation group. When the survivors and non-survivors were analyzed separately, the length of ICU stay in the survivors was significantly greater in the late defecation group than in the early defecation group. The length of mechanical ventilation was not significantly different between the two groups, although it tended to be longer in the late defecation group (Table 4).

**Table 4:** Clinical outcomes

	Early defecation group		Late defecation group		*P* value
ICU mortality, *n* (%)	13	(7)	6	(6)	0.81
Length of ICU stay, days					
All patients	12	(9–19)	16	(10–23)	0.021
Survivors	12	(8–19)	15	(9–23)	0.018
Non-survivors	26	(16–31)	26	(18–34)	0.42
Length of mechanical ventilation, days					
All patients	8	(4–15)	11	(4–19)	0.30
Survivors	7	(3–14)	9	(3–18)	0.39
Non-survivors	18	(11–23)	24	(17–34)	0.28

All data are presented as the median (interquartile range) or *n* (%)

*ICU* intensive care unit

## Discussion

In this study, late defecation was associated with prolonged ICU stay. Consistent with our results, two previous studies showed that the length of ICU stay was significantly longer in patients with late defecation than in those with early defecation [3, 4]. Those studies also showed that the recovery of organ function was impaired in patients with late defecation. In the present study, although the SOFA scores on admission did not differ between the two groups, the scores on ICU day 7 remained high in the late defecation group, whereas the scores in the early defecation group had decreased. Body temperature and serum CRP levels also increased more between admission and ICU day 7 in the late defecation group than they did in the early defecation group. These results indicate that late defecation is associated with sustained organ failure and inflammatory activity.

Infection is the most common cause of inflammation in critically ill patients. As far as we know, this is the first study to assess the potential association between inflammatory markers and constipation in ICU patients. Gacouin et al. [3] reported that acquired bacterial infections were more common in patients with constipation. Furthermore, it has been reported that more than 90 % of ICU patients with infection had at least one episode of infection caused by gastrointestinal bacteria [14]. It is conceivable that impaired peristalsis might cause the progressive translocation of bacteria and endotoxins from the gut into the systemic circulation, stimulating a systemic inflammatory response [15]. Furthermore, an alteration in the microbiota, which is often observed in critically ill patients, is closely related to inflammation. Recent studies have addressed the bi-directional interactions between gut microbiota and the host immune system through functional communication among B cells, T cells, and immunoglobulin A [16, 17]. Other authors suggested the pathways between an alteration of gut microbiota and gastrointestinal dysfunction include direct interactions with the mucosal epithelium, via immunocytes, and via contact to neural endings, resulting in an increase in intestinal permeability, and changes in motility, secretion, blood flow, and mucosal immune activity [18–21]. Consequently, critical illness, inflammation, and defecation are closely related to each other, leading to prolonged ICU stay.

In this study, the independent risk factors for late defecation were late enteral nutrition, sedatives, and surgery; these factors are all related to medical practice. Our finding that late enteral nutrition is a risk factor for late defecation is consistent with a previous report [22]. In fact, in the present study, the median time from admission to the initiation of enteral nutrition was 3 days in the late defecation group compared with 2 days in the early defecation group. Late enteral nutrition itself could cause late defecation by interrupting the mechanical stimulation of peristaltic activity.

The type, dose, and rate of enteral nutrition also affect defecation. Many patients in this study were administered commercially available enteral nutrition either intermittently or continuously through a gastric tube. The nutrient concentration was kept low to start with, and the total amount of energy and the rate of enteral feeding were adjusted daily after evaluating the patient’s condition and the need for energy and water. Normally, enteral nutrition is started at a rate of 5–10 kcal/ideal body weight (in kg)/day and is increased to 20–25 kcal/ideal body weight (kg)/day by ICU day 7. However, because of the variety of feeding regimens used for the patients in this study, we cannot assess the association between quality of enteral nutrition and defecation.

Based on reports demonstrating associations between early/late enteral nutrition and mortality, infectious morbidity, and length of ICU stay, the ASPEN recommends initiating enteral nutrition to critically ill patients within 24–48 h of admission [14, 23, 24]. However, in critically ill patients, early initiation of enteral nutrition is often prevented by peristaltic disabilities of various causes, such as hypoxia and hypotension, which are previously identified risk factors for late defecation [3]. Although early enteral nutrition is not always easy, especially in critically ill patients, the timing of initial enteral nutrition becomes particularly important.

The administration of sedatives was an independent factor for late defecation in this study. In our ICU, the quality of sedation was evaluated using the Richmond Agitation Sedation Scale (RASS). The attending physician needed to set the target RASS score for each patient upon admission to the ICU, and the nurse assessed the RASS every 2–4 h throughout the ICU stay and adjusted the doses of sedatives in accordance with the physician’s recommendations. Half of the patients enrolled in the study were continuously sedated. Propofol and midazolam, which were mainly administered to these patients, adversely affect gastrointestinal function and inhibit bowel motility [25–27]. Moreover, the continuous administration of sedatives generally suppresses the patients’ body activities for several days and is associated with a high incidence of constipation [7]. Therefore, the results of the present study reasonably suggest that the continuous administration of sedatives is a risk factor for late defecation.

Surgery was also identified as an independent risk factor for late defecation in this study. It is well known that abdominal surgery inhibits intestinal peristalsis, but the majority of surgical procedures performed in this study were brain surgery. Although several studies have shown that neurological diseases, such as stroke, influence the occurrence of constipation [7, 28], to our knowledge, there are no reports describing an association between brain surgery and defecation. Although brain surgery per se was not identified as an independent explanatory variable when re-analyzed in the multivariate analysis (*P* = 0.066; data not shown), it is conceivable that brain surgery disturbs gastrointestinal function. In terms of the relationship between central nervous and enteral nervous system, the brain–gut–microbiota axis has been a focus of numerous studies because of its potential functional roles [18–21, 29]. Some studies have demonstrated that brain-derived neurotrophic factor (BDNF), a member of the neurotrophic family, plays an important role in this axis [30, 31]. Interestingly, BDNF shows a close relationship to microbiota. Experimental studies demonstrated that hippocampal expression of BDNF was reduced by modulating the balance of microbiota [30, 32]. Furthermore, BDNF expression in mucosal epithelial and lamina propria cells was reported to be lower in slow-transit constipation patients [33]. According to these findings, we can speculate that alterations in the gut microbiota and reduced BDNF expression following brain surgery contribute to late defecation.

It was reported that opioid use, like sedative use, is associated with late defecation in ICU patients [3, 4, 34]. However, fentanyl, the only opioid administered to the patients in this study, belongs to the class of short-acting narcotics. Therefore, it should have a weak influence on bowel motility. It was also reported that fentanyl increases mesenteric blood flow and has a little effect on gastrointestinal function [35, 36]. These findings support our data showing that fentanyl is not a risk factor for late defecation.

Some researchers have demonstrated that late defecation is associated with a failure of ventilator weaning [1, 5] and prolonged mechanical ventilation [3, 4]. However, our multivariate analysis showed no significant relationship between late defecation and mechanical ventilation. We consider that the non-significant association between these factors was caused by the administration of sedatives. Generally, mechanical ventilation was used in combination with sedatives. In fact, many patients who were mechanically ventilated in this study received sedatives, suggesting that only sedatives or both sedatives and mechanical ventilation are the risk factor(s) for late defecation. Statistically, because the association between sedatives and mechanical ventilation was significant, it is likely that the disadvantageous effect of mechanical ventilation on defecation was comparatively insignificant.

In this study, interventions to promote bowel elimination were initiated earlier in the early defecation group than in the late defecation group. The proportion of patients who underwent such interventions was significantly greater in the late defecation group than in the early defecation group. Because of the nature of this study, it is likely that the physicians prescribed laxatives to patients whose first defecation was delayed. Unfortunately, in the statistical analyses, it was difficult to appropriately adjust for the influence of these interventions on defecation because the timing and choice of intervention were determined by the attending physicians. In fact, when “intervention” was included in the multivariate analysis, it was statistically significant (*P* < 0.001), which indicated that the use of an intervention is not a cause of late defecation but is rather due to delayed defecation. Therefore, we did not include the factor intervention in the multivariate model for predicting the cause of late defecation.

Although we did not demonstrate a clinical implication of intervention in this study, previous studies have shown that the implementation of intervention protocols for constipation reduced the incidence of constipation [37]. van der Spoel et al. showed in a randomized trial that both lactulose and polyethylene glycol were more effective in promoting bowel elimination than a placebo and that the length of ICU stay in patients administered lactulose was significantly shorter than that in the control group [6]. These data suggest that the efficacy of interventions for constipation warrants evaluation in a future study.

We have discussed the risk factors for late defecation and its association with clinical outcomes, and it is difficult to discriminate whether late defecation was a cause of the poor outcomes, a result of medical practice, or reflected the severity of the patients’ conditions because multidirectional factors are involved in the medical course of critically ill patients. However, it is important that physicians know that critically ill patients are at high risk of constipation, which may adversely affect clinical outcomes.

There were several limitations to this study. First, because this was a single-center retrospective study, it included a limited number of patients with heterogeneous backgrounds. Second, the definition of late defecation is still controversial. In this study, we used a cut-off value of 6 days to differentiate between early/late defecation, as in several recent large-scale studies [3, 4, 6, 38], but other studies have used cut-off values of 3 days [1, 5, 22] or 4 days [2, 39]. Third, regarding the analyses of inflammation-related variables, we did not prohibit the use of medications or procedures likely to affect inflammatory markers, particularly corticosteroids, non-steroidal anti-inflammatory drugs, and extracorporeal circulation. Fourth, we did not conduct additional analyses on previously identified risk factors for defecation, such as hypotension and hypoxemia. Although our results must be interpreted carefully, we believe that this study has provided important information to help researchers design prospective studies in the future.

## Conclusions

This study revealed that late enteral nutrition, sedative use, and surgery were the independent risk factors for late defecation in critically ill patients and late defecation was associated with a prolonged ICU stay.

## Abbreviations

APACHE II: Acute Physiology and Chronic Health Evaluation (second revision)
ASPEN: American Society for Parenteral and Enteral Nutrition
BDNF: brain-derived neurotrophic factor
CI: confidence interval
CRP: C-reactive protein
ICU: intensive care unit
IQR: interquartile range
OR: odds ratio
SOFA: Sequential Organ Failure Assessment
